# Prevalence of chronic post-thoracotomy pain in patients with traumatic multiple rib fractures in South Korea: a cross-sectional study

**DOI:** 10.1038/s41598-021-82273-6

**Published:** 2021-01-28

**Authors:** Kun Hyung Kim, Chan Kyu Lee, Seon Hee Kim, Youngwoong Kim, Jung Eun Kim, Yu Kyung Shin, Junepill Seok, Hyun Min Cho

**Affiliations:** 1 Department of Korean Medicine, Pusan National University Hospital, 179 Gudeok-ro, Seo-gu, Busan, 49241 South Korea; 2 Department of Trauma Surgery, Pusan National University Hospital, Busan, South Korea; 3 Trauma Center, Department of Thoracic and Cardiovascular Surgery, Ulsan University Hospital, Ulsan, South Korea; 4 Biomedical Research Institute, Pusan National University Hospital, Busan, South Korea; 5 Department of Trauma and Acute care Surgery, Chungbuk National University Hospital, Cheongju, South Korea; 6 Department of Trauma Surgery, Cheju Halla Hospital, 65 Doryeong-ro, Jeju, 63127 South Korea

**Keywords:** Risk factors, Trauma, Epidemiology

## Abstract

Chronic post-thoracotomy pain is a debilitating condition after traumatic multiple rib fractures and surgery. We aimed to estimate the prevalence of chronic post-thoracotomy pain after traumatic multiple rib fractures in South Korea and explore factors associated with it. From October 2017 to June 2019, a cross-sectional survey of 100 adults, who had undergone thoracotomy due to traumatic fractures of two or more ribs 2 years to 3 months prior to the survey, was conducted in the regional trauma center in South Korea. In total, 80% and 65% patients reported any level and above moderate chronic pain, respectively. Quality of life was mostly below the normative value of the US general population. Forty-six percent patients had restrictive respiratory dysfunction, and 47% and 59% patients were classified as being at risk of above mild-level anxiety and depression, respectively. More than 70% of patients had a current opioid prescription. Multivariable logistic regression analysis showed weak evidence of association between acute, severe postoperative pain and chronic postsurgical pain (adjusted odds ratio 2.4, 95% confidence intervals 0.9 to 6.4). Collectively, chronic post-thoracotomy pain and associated incomplete recovery regarding respiratory, functional, and psychological outcomes were prevalent in patients with traumatic multiple rib fractures in South Korea.

## Introduction

The International Classification of Diseases 11th revision (ICD-11) defines chronic postsurgical pain or chronic traumatic pain as pain that develops or increases in intensity after a surgical procedure or a tissue injury and persists beyond the healing process, i.e., at least 3 months after the initiating event^1^. Patients who undergo surgical fixation of traumatic multiple rib fractures often experience disabling chronic postsurgical pain, which seriously limits activity of daily life and impairs quality of life^2,3^. The prevalence of chronic post-thoracotomy pain (CPTP) ranges from 25 to 91%, showing considerable heterogeneity, which possibly attributed to differences in the underlying pathophysiology, mode of injury if relevant, design of study, methods of classifying pain, and clinical context^3–5^. Given the high prevalence and the detrimental impacts on the functional recovery process and quality of life in the affected patients^6,7^, CPTP after traumatic multiple rib fractures deserve recognition both as a major clinical problem and as a significant public health burden^8,9^. Factors associated with CPTP may include demographic characteristics, such as age, gender, education, and socioeconomic status, and clinical characteristics, such as severity of injury, poly-trauma, comorbid conditions, preoperative pain level and acute postoperative pain level^10–14^. Knowledge on the prevalence of CPTP after traumatic multiple rib fractures and its associated factors are fundamentally important for developing risk-stratification strategies and identifying modifiable risk factors which likely contribute to the management and prevention of CPTP after traumatic multiple rib fractures and the informed decision-making process regarding appropriate pain management by relevant stakeholders (i.e., patients, caregivers, healthcare professionals and policymakers)^3,9,15–17^. Currently, such information remains unclear in the South Korean population. To address this gap of knowledge, the study aimed to estimate the prevalence of chronic post-thoracotomy pain after traumatic multiple rib fractures in the South Korean setting and explore factors associated with it.

## Results

**Figure 1:**
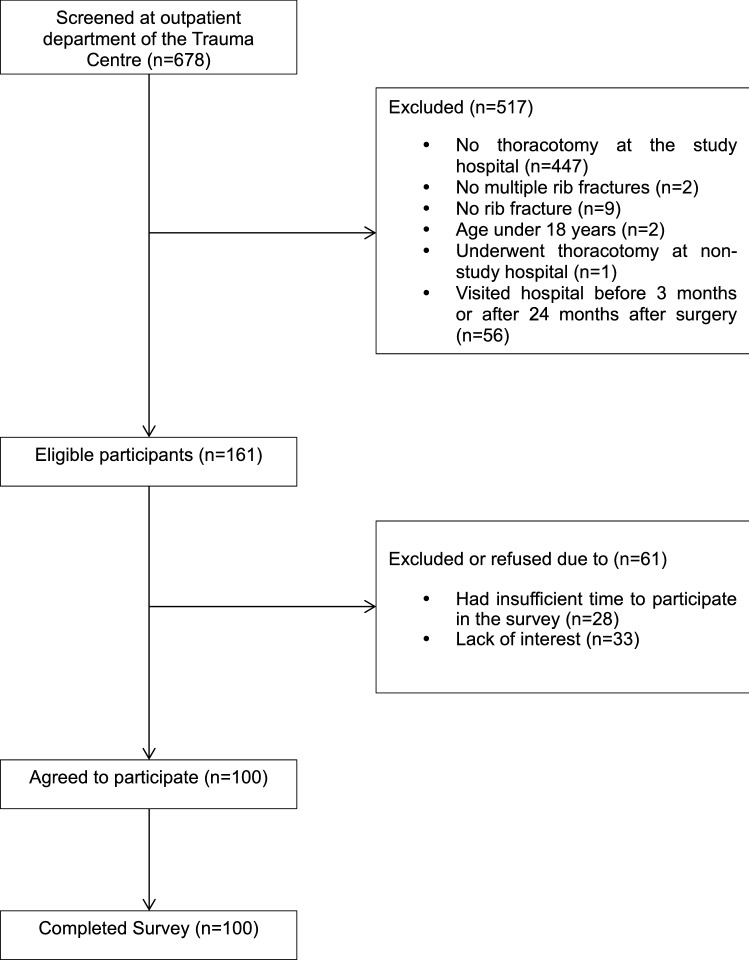
Patient flowchart.

The study was performed at outpatient clinics in the Regional Trauma Center at Pusan National University Hospital. Among the 678 outpatients, 161 eligible patients were asked to participate in the survey to achieve the anticipated 100 participant’s responses (i.e., 62% of eligible patients being participated in the survey) (Fig. 1).

### Demographic and clinical characteristics

There was no difference in the demographic, clinical characteristics and comorbidities at the time of injury (Supplementary Table S1) between patients with and without chronic pain (Table 1). The mean age of the survey respondents was 56.7 (SD 12.1) years. Most participants were male (82%). All patients were classified as having serious or above-level chest trauma using the AIS chest criteria, and nearly a half of patients were polytraumatised. The most common surgical incision site was the lateral chest wall (89%). The time of the survey from the surgery was 3 to 6 months in about half of patients. Other detailed demographic and clinical characteristics are provided in Table 1.

**Table 1:** Descriptive and bivariate analysis of demographic and clinical characteristics of the survey participants.

Variables	Categories	Total (n = 100)	No chronic pain (n = 35)	Chronic pain (n = 65)
Age		56.7 (12.1)	55.0 (11.3)	56.2 (12.2)
Female		18 (18%)	5 (14.3%)	13 (20%)
Body mass index (kg/m^2^)		24.1 (3.3)	24.5 (3.8)	24.0 (3.0)
Smoking	Active	23 (23%)	6 (17.2%)	17 (26.2%)
	Past/never	26 (26%)	9 (25.7%)	17 (26.2%)
Alcohol	None	30 (30%)	6 (17.1%)	24 (37.0%)
	Less than 4 times/months	41 (41%)	19 (54.3%)	22 (33.8%)
	2 or more times/weeks	29 (29%)	10 (28.6%)	19 (29.2%)
Education	Middle school or lower	30 (30%)	10 (28.6%)	20 (30.8%)
	High school	43 (43%)	15 (42.8%)	28 (43.1%)
	College or university	27 (27%)	10 (28.6%)	17 (26.1%)
Poly-trauma		47 (47%)	18 (51.4%)	29 (44.6%)
ISS score		21 [17, 27]	22 [17, 29]	20 [17, 27]
ISS ≥ 16		82 (82%)	29 (82.9%)	53 (81.5%)
AIS chest score	Serious	64 (64%)	21 (60.0%)	43 (66.2%)
	Severe or critical	36 (33%)	14 (37.1%)	22 (30.8%)
Number of fractured ribs		8.5 [4, 15]	8.0 [6.0, 9.0]	8.0 [6.0, 11.0]
Location of surgical incision	Anterior	21 (21%)	4 (11.4%)	17 (26.2%)
	Lateral	89 (89%)	32 (91.4%)	57 (87.7%)
	Posterior	49 (49%)	18 (51.4%)	31 (47.7%)
	Bilateral	6 (6.1%)	1 (2.9%)	5 (7.8%)
Bilateral fracture		39 (39%)	11 (31.4%)	28 (43.1%)
Fracture of sternum		10 (10%)	3 (8.6%)	7 (10.8%)
Degree of dislocation	None/mild	25 (25%)	7 (20%)	18 (28%)
	Moderate	46 (46%)	15 (43%)	31 (48%)
	Severe	29 (29%)	13 (37%)	16 (24%)
Removal of fixation plate		8 (8%)	2 (5.7%)	6 (9.4%)
Preoperative chest tube insertion		63 (63%)	25 (71.4%)	38 (58.5%)
Tube insertion period (day)		10 [7, 13]	11 [7, 14]	9 [7, 13]
Postoperative complication^a^		2 (2%)	1 (2.9%)	1 (1.5%)
Mode of injury	Fall	36 (36%)	11 (31%)	25(38%)
	Vehicle injury	50 (50%)	19 (54%)	31(48%)
	Crush	14 (14%)	5 (14%)	9(14%)
Time from surgery (months)	3 to 6 months	50 (50%)	16 (45.7%)	34 (52.3%)
	6 to 12 months	30 (30%)	13 (37.1%)	17 (26.2%)
	≥ 12 months	20 (20%)	6 (17.2%)	14 (21.5%)
Acute severe pain during admission		35 (35.4%)	8 (22.9%)	27 (42.2%)
Intravenous PCA		88 (88%)	27 (77.1%)	61 (93.9%)
Epidural PCA		39 (39%)	17 (48.6%)	22 (33.9%)

AIS; abbreviated injury score, ISS; injury severity score, PCA; patient controlled analgesia.

^a^Postoperative complications include superficial wound infections in both group.

### Prevalence of chronic post-thoracotomy pain after traumatic multiple rib fractures

Eighty percent patients self-reported chronic post-thoracotomy pain after traumatic multiple rib fractures. Sixty-five percent patients had moderate or severe level of pain in any of the four situations (at rest, during movement, lying down, and while coughing). The proportion of patients with neuropathic pain was 51%. (Table 2).

**Table 2:** Prevalence and characteristics of chronic pain.

Variables	N = 100 (95% CI)
**Self-reported pain within the recent week**	
Yes	80 (80%, 71% to 87%)
**Pain intensity**	
At rest	3.5 (3.0, 4.0)
During movement	4.0 (3.5, 4.6)
While coughing	3.7 (3.1, 4.4)
Lying down	5.3 (4.8, 6.0)
**Above moderate pain**	
At rest	36 (36%, 27% to 46%)
At movement	39 (39%, 29% to 49%)
Coughing	40 (40%, 30% to 50%)
Lying down	57 (57%, 47% to 67%)
Overall	65 (65%, 55% to 74%)
Neuropathic pain	51 (51%, 41% to 61%)

CI: confidence interval.

### Anxiety and depression

Forty-seven percent patients were classified as having at least mild-level anxiety. More than half of the patients (56%) were classified as being depressive. Almost one in 10 patients was found to have a risk of severe anxiety and/or depression (Supplementary Table S2). There was 9 times higher odds of having above-mild level anxiety in patients with chronic pain compared to those without chronic pain, when adjusted for age, sex, level of education, current smoking, and injury severity (n = 100; adjusted OR 9.1, 95% CI 2.9 to 28.5). There was 12 times higher odds of having above-mild level depression in patients with chronic pain compared to those without chronic pain, when adjusted for the same covariates (n = 100; adjusted OR 12.6, 95% CI 4.3 to 37.3).

### Pulmonary function

Three patients refused the test due to discomfort of the process. The average FEV1 in patients who completed the test was 77% compared to the reference value. Nearly half of the patients (46%) who had undergone the pulmonary function test showed a restrictive respiratory pattern. A small number of patients (8%) showed an obstructive respiratory pattern. There was no difference in the respiratory function parameters between patients with and without chronic pain, when adjusted for age, sex, level of education, current smoking status, and injury severity scale (≥ 16 or not) (Supplementary Table S3).

### Quality of life

In all the 10 health domains, the average or median value of SF-12_v_2 scores were below those of the 2009 US general population normative value (i.e., score of 50 in each domain)^4^. Patients with chronic pain had significantly worse quality of life than those without chronic pain in both physical and mental domains, when adjusted for age, sex, level of education, current smoking status, and ISS score (Supplementary Table S4).

### Prescription of opioids and other medication

At the time of survey, 74% patients had a current opioid prescription. Gabapentinoid was prescribed in 63% patients. Twenty-two percent patients were using non-steroidal anti-inflammatory drugs (NSAIDs) and 21% psychotropic drugs. Acetaminophen was prescribed in one patient (Supplementary Table S5).

### Association between acute severe pain and presence of chronic post-thoracotomy pain

In a bivariate analysis, most of the demographic and clinical characteristics, except experience of intravenous PCA during admission, were not associated with chronic pain (Table 1). Severe acute-phase pain measured before discharge showed marginal association with increased risk of chronic pain (OR 2.5, 95% CI 1.0 to 6.3). Age at the surgery, sex, level of education, current smoking status, ISS score, and AIS chest score were included in the multivariable logistic regression model as a priori confounders, regardless of statistical significance in the bivariate analyses. Experience of intravenous PCA was included in the model based on the statistical significance and as a potential source of confounding by indication. When adjusted for age, sex, level of education, current smoking status, ISS score, AIS chest score, and experience of IV-PCA, there was similar association between severe acute-phase pain before discharge and chronic pain (aOR 2.4, 95% CI 0.9, 6.4) (Table 3). To explore whether experience of IV-PCA was a confounder by indication, stratified analysis by acute severe pain was conducted. Experience of IV-PCA has lost significant association with chronic pain in both strata (strata of acute severe pain; aOR 15.0; 95% CI 0.6, 377.7 and strata of no acute severe pain; aOR 3.5; 95% CI 0.7, 17.9). There was no significant change in the direction, magnitude, and uncertainty of the effect estimates, when adjusted for age, sex, ISS score, preoperative chest tube insertion, bilaterality of rib fractures and fractures of rib cartilage (OR 2.4, 95% CI 0.9, 6.4) (data not shown).

**Table 3:** Factors associated with chronic post-thoracotomy pain after traumatic multiple rib fractures (n = 99).

	uOR (95% CI)	aOR (95% CI)
**Age (year)**		
≤ 44	1	1
45 to 54	1.6 (0.5, 5.6)	1.5 (0.4, 5.8)
55 to 64	1.7 (0.5, 5.4)	2.2 (0.5, 8.8)
≥ 65	1.4 (0.4, 4.3)	1.8 (0.4, 7.7)
**Sex**		
Male	1	1
Female	1.5 (0.5, 4.6)	1.5 (0.4, 5.1)
**Higher level of education (categorized)**		
Middle school or lower	1	1
High school	0.9 (0.3, 2.5)	1.4 (0.4, 4.6)
College or university	0.9 (0.3, 2.5)	1.4 (0.4, 5.2)
**Smoking**		
Past/never	1	1
Currently smoking	1.7 (0.6, 4.8)	2.1 (0.6, 6.8)
**ISS score**		
< 16	1	1
≥ 16	0.9 (0.3, 2.7)	0.7 (0.2, 2.6)
**AIS chest**		
3	1	1
≥ 4	0.8 (0.3, 1.8)	0.8 (0.3, 2.1)
**Severe pain before discharge**		
No	1	1
Yes	2.5 (1.0, 6.3)	2.4 (0.9, 6.4)
**Experience of IV-PCA**		
No	1	1
Yes	4.5 (1.3, 16.3)	5.5 (0, 8.4)

Values of unadjusted estimates are from bivariate analysis between each factor and presence of moderate to severe chronic pain.

uOR: unadjusted odds ratio; aOR: adjusted odds ratio; CI: confidence interval.

## Discussion

We found the significantly high proportion of participants with CPTP. Anxiety and depression were also prevalent. Overall, quality of life was worse than the known normative value in the US general population and significantly worse in patients with CPTP than in those without CPTP in several health domains. Nearly half of the patients were classified as having restrictive-pattern respiratory dysfunction, suggesting the prevalent respiratory dysfunction, regardless of CPTP. Prolonged use of opioids was found in over two thirds of the participants. There was some weak evidence of an association between the experience of acute severe pain during admission and with CPTP, although the wide confidence intervals might imply insufficient power to detect their association. Collectively, our findings highlight the significant burden of CPTP, psychological distress, lowered quality of life, delayed respiratory function, and prolonged use of opioids in patients who underwent thoracotomy after traumatic multiple rib fractures in South Korea.

In our study, the prevalence of self-reported CPTP was 80% when classified as any level of pain and 65% when classified as moderate to severe level. Prevalence of pain after rib fractures and subsequent conservative management was 59% at 2 months post-injury^18^. Two small studies conducted in Europe and the US showed lower prevalence of chronic pain (16%) among respondents in patients who had undergone stabilizing surgery of multiple rib fractures and/or flail chests^19,20^. In a recent narrative review, the incidence of chronic pain after thoracic surgery, including either open or video-assisted thoracotomy (VAT), remained stable at around 50% over time^21^. Inconsistencies between previous findings may be attributable to clinical and methodological heterogeneity, including but not limited to, difference in definition of chronic pain, time-point of measuring pain, research design (e.g., cross-sectional survey or cohort study) or methods of data collection (e.g., face-to-face survey or telephone interview). Currently, no clear explanation for inconsistency of evidence seems available at the moment which justifies the further systematic investigation on the risk of CPTP in patients after traumatic multiple rib fractures.

The prevalence of neuropathic pain among patients who reported a moderate or severe level of chronic pain was consistent with that reported in a previous systematic review^22^, which reported that the prevalence of probable or definitive neuropathic pain was 66% in patients with persistent CPTP. Whether the prevalence of neuropathic pain after thoracic surgery related to trauma differs from those for non-traumatic etiologies needs further investigation.

We found no significant association between CPTP and pulmonary function test results. It is of note that the overall proportion of patients with restrictive respiratory dysfunction was almost 50% regardless of chronic pain, which may imply that the burden of respiratory dysfunction is common in patients who had received thoracotomy. In a single-center audit in South Korea involving 72 patients with traumatic multiple rib fractures who had PFT results 6 to 23 months from discharge, restrictive respiratory dysfunction (i.e., FVC < 80% of predicted values) was present in 42% patients^23^, although these results need careful interpretation as respiratory function was measured at variable timepoints after discharge.

We found that quality of life was consistently lower in all domains than that of the reference population value and tended to be significantly lower in patients with chronic pain than in those without chronic pain. Our findings resonate with previous studies conducted in Australia, reporting that multiple rib fractures are associated with lowered quality of life over 2 years post injury in patients with multiple rib fractures regardless of surgical fixation surgery^7,24^. Patients without chronic pain also showed impaired quality of life in terms of PCS scores. Claydon reported that patients with multiple rib fractures faced challenges with rehabilitation throughout the entire recovery journey, which mainly arose from difficulties with coping with chronic pain and long-term restriction of lifestyle and functional abilities^6^. These findings suggest that limited physical function may present independent of chronic pain and can be an important outcome that deserves attention from healthcare professionals. Since chronic pain and poor quality of life may mutually affect each other in an intertwined way and not necessarily be captured by the questionnaire-based assessments^25^, triangulation of our findings by both quantitative and qualitative studies would help understand the potential complexity of association between chronic pain and quality of life in patients with chronic post-thoracotomy pain after multiple rib fractures.

A recent systematic review found that the prevalence of continued opioid use after trauma or surgery was 4.3% (95% CI 2.3% to 8.2%)^26^. The US population-based cohort study revealed that the risk of opioid use 90 to 180 days after various elective surgery was 7.1%^27^. In our findings, a much higher proportion of patients were classified as prolonged users of opioids, which might be associated with a different clinical context and distribution of risk factors for chronic use of opioids. However, we could not exclude risk of overestimation as prescribed opioids might have not been actually consumed by patients. The prescribing pattern of physicians and pain-coping behaviors of patients are also related factors which should be explored in larger prospective studies.

Acute pain intensity is known to be associated with chronic pain in patients with rib fractures^2,28^. We found some weak evidence of an association between the experience of severe pain in acute stage trauma and CPTP. Heterogeneity in clinical, sociodemographic, and methodologic aspects might have contributed to the observed discrepancy. For instance, previous studies were mostly regarding patients who had undergone elective surgery^28^, in which the underlying pathophysiology, the degree of concurrent neural and bony tissue injury, and psychological impacts of traumatic events may be considerably different from those who had received non-elective surgery after trauma. Nurse records of acute-stage pain might have been documented inaccurately and may have affected the study findings. Time from surgery was various in our cross-sectional study, which may have contributed to the misclassification of CPTP. Therefore, our findings require careful interpretation and should not be regarded as confirmative evidence of no association between acute and chronic pain. Observed association between use of IV-PCA and chronic pain is possibly due to confounding by indication, as stratified analyses revealed no significant association between them.

To the best of our knowledge, this is a first descriptive cross-sectional survey investigating the prevalence of CPTP in patients with traumatic multiple rib fractures in South Korea. A dedicated, trained researcher measured outcomes using validated scales and instruments. Almost all patients provided results of the pulmonary function test, which may triangulate the validity of our findings on the impaired physical functions assessed by the subjective questionnaire. There were a few missing responses among the survey participants, which limits the risk of bias due to the non-response. The power of the study was enough to test the null hypothesis that the CPTP in patients with multiple rib fractures would be 25%. We adhered to the reporting guideline of observational studies in the design and reporting of the survey.

Limitations are as follows: This is a cross-sectional study, which could not distinguish between prevalent (existing) pain and incident (new-onset) pain^29^. It remains unclear whether trauma or surgery were solely responsible for chronic pain due to the absence of data regarding pain before trauma. Data acquisition was available only in patients who physically presented at the outpatient clinic. This may have introduced selection bias if the patients who have visited the hospital and underwent survey were systematically different from those who did not visit the hospital after surgery. Other logical barriers such as time constraints or unaffordability of the hospital visit should also be considered as potential source of non-presentation. In our study, the timepoint of measuring chronic pain was inconsistent, which would be a potential source of variance of the prevalence estimation. The survey was conducted in the single regional trauma center; therefore, the respondents may not represent the study population (i.e., the respective patients in the South Korea) and our estimations may not reflect the true population prevalence of chronic post-thoracotomy pain after traumatic multiple rib fractures. Although we asked participants to rate their pain focusing on the chest area, patients who were poly-traumatized and had injuries other than in the chest may not differentiate the pain limited in the chest region and the perceived overall bodily pain. Therefore, we could not exclude the risk of bias due to misclassification of CPTP. Findings on clinical outcomes should serve exploratory purposes due to the risk of type 1 error when multiple analyses were performed.

To overcome aforementioned limitations, prospective investigation of incidence of CPTP in patients undergoing thoracotomy after chest trauma is highly warranted. Standard methodology and timepoints for measuring pain and other patient-relevant outcomes as well as its transparent reporting are necessary to produce comparable quality of data, which would be used for the reliable estimation of CPTP following traumatic multiple rib fractures. Burden of opioid and psychotropic drug use in this population should be addressed in further larger-scale studies, because of their potential harms when used long-term. Surveillance of chronic pain, quality of life, psychological comorbidity, and respiratory function after surgery to inform researchers and clinicians for providing the best-available care to patients are required. Return to work or social activity which reflects occupational and social aspects of recovery were not measured in this study and need to be addressed in future studies^30^. Valid ascertainment methods of acute-stage pain in patients undergoing surgery after traumatic multiple rib fractures should be developed, as routine clinical documents collected for non-research purposes may not be a reliable source of research data.

## Conclusion

Chronic post-thoracotomy pain was prevalent in patients with traumatic multiple rib fractures in South Korea. Impaired respiratory function, suboptimal quality of life, depressive mood, and possible prolonged use of opioids were also common. There was some weak evidence of an association between acute postoperative pain and CPTP. Further prospective and population-representative studies are required for the reliable estimation of CPTP in South Korea.

## Methods

### Study hypothesis

We hypothesized that the estimated risk of chronic post-thoracotomy pain after traumatic multiple rib fractures is 25%, the known baseline risk in patients who have undergone elective thoracic surgery for non-traumatic etiologies^3^. A secondary hypothesis was that acute-phase severe pain after injury is a significant risk factor of chronic persistent pain, based on previous studies that have highlighted the association between acute-stage severe pain and chronic post-thoracotomy pain after multiple rib fractures^2,31^.

### Study design and setting

We used a cross-sectional design, which is less complex than other designs and appropriate enough for estimating the prevalence of chronic pain when limited information is available for the topic. We defined the study population as adults dwelling in South Korea who underwent thoracotomy (i.e., open reduction and internal fixation of fractured ribs using screws and plates) 2 years to 3 months prior to the survey due to multiple rib fractures associated with blunt chest trauma. The survey was conducted in the regional Trauma center in Pusan National University Hospital (PNUH), which is located in the center of Busan City and covers traumatic injury cases in Busan City and the outskirts of South Gyeongsang Province from October 10, 2017 and June 11, 2019. The PNUH Trauma center was established on November 9, 2015. The cumulative number of patients with multiple rib fractures admitted and discharged in the center from inception to December 31, 2019 is approximately 1694.

### Eligibility criteria

Adults aged ≥ 19 years who had undergone thoracotomy due to traumatic multiple rib fractures 2 years to 3 months prior to the survey were included. To be eligible, patients should have a diagnosis of fractures on two or more ribs (code S22.4) and/or flail chest (code S22.5) according to the International Classification of Diseases, 10th Revision^32^. Patients who were unable to respond to the questionnaire with or without assistance of the caregiver or the researcher for reasons such as cognitive problems or only caregivers were present in the hospital were excluded in the survey.

### Data collection

A structured data collection sheet was developed through discussion between authors. A dedicated, trained research assistant (YKS), who had not been involved in surgery or postoperative care, asked the patient about demographic characteristics and clinical outcomes via a face-to-face interview to avoid response bias. For brevity of the survey process, information available on the electric medical record (EMR) were not asked from the patient; it was extracted from the record by the research assistant or the study trauma surgeons. In most cases, approximately 10 to 15 min were required for the data collection in an interview. Pulmonary function test without bronchodilator administration using spirometry was performed by experienced independent technician assessors who were not aware of the chronic pain status in the respiratory disease center in the PNUH on the same day of the survey.

### Measurement of health outcomes

#### Self-reported pain at any level and pain intensity

Each patient was asked whether he/she felt any level of pain within the recent week. Patient who reported pain were asked to rate the average pain intensity felt at rest, during movement, lying down, and while coughing on the numeric rating scale (NRS) of 0 (no pain at all) to 10 (worst pain imaginable ever) scores. Patients who expressed pain NRS scores of ≥ 4 points for any of the four situations were considered to have moderate-level chronic pain (i.e., the outcome variable in the multiple logistic regression analysis).

#### Neuropathic pain

To screen the patients with neuropathic pain, we administered a validated Korean version of the Leeds Assessment of Neuropathic Symptoms and Signs (LANSS) pain scale to patients who expressed any level of pain in the recent week^33^. It comprises of 5 items for pain and 2 for sensory testing, and the score ranged from 0 to 24 points. Patients with scores ≥ 12 points were considered to have a neuropathic mechanism underlying their pain in the chest area^33^. If a patient reported no pain, the scale was not administered and the level of neuropathic pain was scored as zero. A dedicated trained research assistant (YKS) performed the procedures and assessed the patients.

#### Quality of life

The Korean version of SF-12 Version 2 (SF-12_v_2) Health Quality of Life questionnaire was used to assess the quality of life in the recent 4 weeks^34^. This is a validated scale to measure general health quality of life in the general population of South Korea. Scores of health domain scales and component summary measures were standardized as *T*-scores using a metric with a mean of 50 and a standard deviation of 10 based on the 2009 U.S. general population samples, as indicated in the official user manual of SF-12_v_2^35^. Standardized scores above and below 50 are above and below the average in the 2009 U.S. general population^35^.

#### Self-reported depression and anxiety

The Korean version of Hospital Anxiety and Depression Scale (HADS) was used to measure self-reported depression and anxiety in the survey participants^36^. Patients were asked to rate their condition at the time of survey. Anxiety and depression subscale scores were categorized into no (0 to 7 points), mild (8 to 10), moderate (11 to 14), and severe (15 to 21) degree of anxiety and depression, respectively^37^.

#### Respiratory function

We measured forced vital capacity of predicted value (FVC%), forced expiratory volume in one second of predicted value (FEV1%), and the ratio of FEV1% to FVC%. Restrictive respiratory dysfunction was defined as having FEV1/FVC ratio ≥ 0.7 and FVC% < 80% of predictive value. Obstructive respiratory dysfunction was defined as having FEV1/FVC ratio < 0.7 and FVC% ≥ 80% of the predictive value. Mixed obstructive-restrictive respiratory dysfunction was defined as having both FEV1/FVC ratio < 0.8 and FEV1% < 80% of the predictive value^38^.

### Measurement of acute-stage pain intensity (exposure variable)

Acute-stage pain intensity was defined as the severity of pain experienced by patients from the traumatic event to discharge from the thoracic trauma ward. As participants were unlikely to recall the intensity of acute-stage pain reliably, we used the highest pain intensity measured by a 0 to 10 NRS from the trauma-ward nurse records in EMR on admission to the thoracic trauma ward as a proxy of acute-stage pain intensity. Acute, severe pain, a dichotomized variable defined as pain rated ≥ 6 points, was generated as an exposure variable in multiple logistic regression analysis. Preoperative pain intensity measured in the emergency room was not used to avoid inaccurate and inconsistent pain assessment.

### Confounders, effect modifiers, and covariates

A number of sociodemographic, clinical, and radiological covariates were obtained either in the survey questionnaire or the EMR. A priori confounders were age at the surgery, sex, level of education as a proxy of socioeconomic status, current smoking status, injury severity scale (ISS) score ≥ 16, and abbreviated injury scale (AIS) score ≥ 4 on chest as a proxy of severe traumatic injury. Other potentially relevant factors included bilaterality of the rib fractures, injury of the costal cartilages, preoperative chest tube insertion, and day of preoperative chest tube insertion. We tried to examine effect modification by sex, although this was not possible due to the smaller number of female patients. Potential confounders and effect modifiers were determined by the trauma surgeons and the researchers after literature reviews and discussions. Alcohol consumption was considered a behavioral covariate. Clinical covariates included the location of rib fractures (anterior, lateral, posterior, unilateral, or bilateral), the number of fractured ribs, degree of dislocation (none to mild, moderate, or severe) determined by trauma surgeons (KYW and CKL), removal of the plates, injury of costal cartilage, mode of injury (such as fall, vehicle accident, or crushing), time elapsed from surgery (months), length of hospital stay (days, from the ER admission to discharge from the thoracic trauma ward), polytrauma, comorbidities identified at the injury, insertion of preoperative chest tube, period of chest-tube insertion, experience of epidural or intravenous patient-controlled analgesia (PCA) which were indicative for patients reporting acute severe postoperative pain, and adverse events or complications after surgery. Patients with multiple rib fractures but not having injuries of other body parts determined by AIS score, were considered to have isolated rib fractures (i.e., AIS score of 1). Patients who had multiple rib fractures and injuries in the other parts of the body (i.e., AIS score ≥ 2 in each of the other parts) were regarded as having polytrauma.

### Statement of research ethics

The Pusan National University Hospital ethics committee approved the survey (Approval number: H-1709-013-058). The research was performed in accordance with the Declaration of Helsinki and the guideline for Good Clinical Practice (GCP). All participants provided written informed consent. The prospective study registration number is KCT0002839 (accessible at https://cris.nih.go.kr/cris/index.jsp).

### Sample size calculation

The anticipated recruitable number of participants was regarded as 100 at the time of study design, based on the logical constraint rather than formal sample size calculation. As limited information was available for the prevalence of chronic post-thoracotomy pain in patients with blunt chest trauma, we aimed to collect data for exploratory analyses and a future formal study. Given the known prevalence (≥ 25%) of chronic post-thoracotomy pain in patients with non-traumatic events^3^, the null hypothesis was that the prevalence of chronic post-thoracotomy pain in patients with blunt chest trauma in South Korea is 25%. When 5% Type 1 errors and 20% Type 2 errors were allowed for one-sample proportion test of the null hypothesis, the estimated expected prevalence in the target population was 37.6%.

### Statistical analysis

For descriptive data, the number and proportion of participants with the outcome of interest were presented. The chi-square test or Fisher’s exact test was used for categorical variables as appropriate. Continuous data were presented as mean ± standard deviation and/or 95% confidence interval. Non-parametric tests were performed, and median and interquartile range were presented when data were non-normally distributed. Binomial analysis was performed to test crude association between the presence of chronic post-thoracotomy pain (i.e., outcome) and confounders and covariates. Multivariable logistic regression was performed using a forward-stepwise approach to identify an association between history of acute severe pain and chronic post-thoracotomy pain, adjusted for a priori confounders and covariates, which showed statistical significance with chronic pain in bivariate analysis. Multi-collinearity issue was considered when building the regression model. Missing values in the health outcomes or exposure were not imputed. There was one missing value of acute pain intensity, and the record was excluded from the multiple logistic regression analysis.

## Supplementary Information

Supplementary Information.

## Data Availability

The questionnaire and datasets generated during and/or analyzed during the current study are available from the corresponding author on reasonable request.
