# Supplementary material for: Prevalence of chronic post-thoracotomy pain in patients with traumatic multiple rib fractures in South Korea: a cross-sectional study
Source: Sci Rep. 2021 Jan 28;11:2615. doi: 10.1038/s41598-021-82273-6 (PMC7844269; doi:10.1038/s41598-021-82273-6)
Supplement: Supplementary file 1 — Supplementary Information. [file 41598_2021_82273_MOESM1_ESM.pdf]

Title page

Prevalence of chronic post-thoracotomy pain in patients with traumatic multiple rib fractures in South Korea: a cross-sectional study

Kun Hyung Kim[1*], Chan Kyu Lee[2], Seon Hee Kim[2], Youngwoong Kim[3], Jung Eun Kim[4], Yu Kyung Shin[4], Junepill Seok[5] and Hyun Min Cho[6*]

[1]Department of Korean Medicine, Pusan National University Hospital, Busan, South Korea

[2]Department of Trauma Surgery, Pusan National University Hospital, Busan, South Korea

[3]Department of Trauma Surgery, Ulsan University Hospital, Ulsan, South Korea

[4]Biomedical Research Institute, Pusan National University Hospital, Busan, South Korea

[5]Department of Trauma Surgery, Wonkwang University Hospital, Jeonbuk, South Korea

[6]Department of Trauma Surgery, Cheju Halla Hospital, Jeju, South Korea

* Two authors equally contributed to the article.

Financial Disclosure: None

Running title: Prevalence of chronic post-thoracotomy pain after traumatic multiple rib fractures

Keywords: wound and injuries, multiple trauma, critical pathways, multidisciplinary care, pain

Co-corresponding authors:

*Kun Hyung Kim, KMD, PhD

Department of Korean Medicine, Pusan National University Hospital, 179 Gudeok-ro, Seo-gu, Busan 49241, South Korea

Tel: +82-55-360-5971

Fax: +82-55-360-5518

E-mail: pdchrist@gmail.com

*Hyun Min Cho, MD, PhD

Department of Surgery, Cheju Halla General Hospital, 65 Doryeong-ro, Jeju 63127, South Korea

Tel: +82-64-740-5000

E-mail: csking1@daum.net

Funding: This research was supported by a grant from the Korea Health Technology R&D Project through the Korea Health Industry Development Institute (KHIDI), funded by the Ministry of Health & Welfare, Republic of Korea (grant number: HI17C1284) The funding body has no role in the study design and will not play any role during its execution, analyses, interpretation of the data, or decision to submit the report for publication.

Supplementary Table S1. Descriptive analysis of comorbid conditions at the time of injury of the survey participants

Comorbidity	Total (n=100)	No Chronic pain (n=35)	Chronic pain (n=65)
None	44 (44.0%)	17 (48.6%)	27 (41.6%)
Yes	56 (56.0%)	18 (51.4%)	38 (58.5%)
Hypertension	24 (24.0%)	8 (22.9%)	16 (24.6%)
Diabetes	10 (10.0%)	2 (5.7%)	8 (12.3%)
Musculoskeletal diseases	16 (16.0%)	4 (11.4%)	12 (18.5%)
Urologic diseases	4 (4.0%)	2 (5.7%)	2 (3.1%)
Stroke	2 (2.0%)	2 (5.7%)	0 (0.0%)
Cancer	2 (2.0%)	1 (2.9%)	1 (1.5%)
Chronic heart diseases	4 (4.0%)	1 (2.9%)	3 (4.6%)
Chronic respiratory diseases	2 (2.0%)	1 (2.9%)	1 (1.5%)
Chronic kidney diseases	1 (1.0%)	0 (0.0%)	1 (1.5%)
Psychiatric diseases	3 (3.0%)	2 (5.7%)	1 (1.5%)
Gastrointestinal diseases	3 (3.0%)	1 (2.9%)	2 (3.1%)
Other conditions	11 (11.0%)	3 (8.6%)	8 (12.3%)

Supplementary Table S2. Self-reported anxiety and depression*

Variables	N=100 (%)	No pain (n=35)	Pain (n=65)	P-value
Anxiety				
None	55 (55%)	29 (82.8%)	26 (40.0%)	<0.001
Mild	28 (28%)	3 (8.6%)	25 (38.5%)	
Moderate	6 (6%)	2 (5.7%)	4 (6.1%)	
Severe	11 (11%)	1 (2.9%)	10 (15.4%)	
Depression				
None	44 (44%)	27 (77.1%)	17 (26.2%)	<0.001
Mild	22 (22%)	5 (14.3%)	17 (26.2%)	
Moderate	24 (24%)	2 (5.7%)	22 (33.8%)	
Severe	10 (10%)	1 (2.9%)	9 (13.8%)	

*: measured by Hospital anxiety and depression scale.

P-value by Fisher’s exact test

Supplementary Table S3. Pulmonary function test

	N	Mean (95% CI) or n (%)	No Pain (n=35)	Pain (n=62)	MD or OR (95% CI)
FVC (%)	97	80.1 (77.2 to 83.1)	81.2 (14.5)	79.5 (14.7)	-3.3 (-9.0, 2.4)
FEV1 (%)	97	77.2 (74.0 to 80.5)	80.0 (15.7)	75.7 (16.0)	-5.2 (-11.7, 1.3)
FEV1/FVC (%)	97	97 [91, 103]	98.2 (8.4)	95.1 (12.1)	-2.3 (-6.6, 2.1)
Restrictive pattern	97	44 (45%, 35% to 56%)	14 (40%)	31 (48%)	1.9 (0.7, 4.9)
Obstructive pattern	97	0 (0%)	0 (0%)	0 (0%)	Not estimatable
Obstructive-restrictive pattern	97	1 (8%)	0 (0%)	1 (2%)	Not estimatable

MD, mean difference; SD, standard deviation; FEV(%), forced vital capacity of predicted value; FEV1(%), forced expiratory volume in one second of predicted value.

Age(group), sex, smoking, education (group), injury severity scale score (group), abbreviated injury scores chest (group) was adjusted for group comparison.

Supplementary Table S4. Quality of Life

Domain	N	Total (n=100)	No pain (n=35)	Pain (n=75)	Mean Differences (95% CI)
Physical component summary	100	38.4 (9.0)	43.0 (8.2)	35.9 (8.4)	-6.7 (-9.9, -3.4)
Mental component summary	100	44.8 (12.3)	50.2 (10.1)	41.8 (12.5)	-8.4 (-13.1, -3.6)
Physical functioning	100	38.5 (9.5)	42.7 (8.8)	36.2 (9.1)	-6.1 (-9.5, -2.7)
Role physical	99	37.6 (10.0)	42.3 (10.4)	35.1 (8.9)	-6.5 (-10.5, -2.6)
Bodily pain	99	39.4 (10.7)	47.2 (8.3)	35.2 (9.5)	-12.3 (-16.1, -8.5)
General health	100	40.4 (11.0)	45.2 (10.7)	37.9 (10.4)	-6.7 (-11.2, -2.3)
Vitality	100	44.1 (11.5)	48.8 (11.3)	41.2 (10.9)	-6.9 (-11.4, -2.3)
Social functioning	100	44.8 (11.4)	48.5 (9.9)	42.8 (11.8)	-5.1 (-9.8, -0.4)
Role emotional	100	40.2 (12.8)	45.9 (10.8)	37.2 (12.8)	-8.9 (-13.9, -3.8)
Mental health	100	42.7 (12.6)	49.5 (9.9)	39.1 (12.4)	-10.4 (-15.1, -5.7)

Values are from norm-based scoring (NBS) based on the U.S. general population normative sample 2009. Recall-period: 4 week.

Age(group), sex, smoking, education (group), injury severity scale score (group), abbreviated injury scores chest (group) was adjusted for group comparison.

Supplementary Table S5. Use of opioids and other medication

	N	n (%)	No Pain (n=35)	Pain (n=65)	OR (95% CI)
Opioid	100	74 (74%)	25 (71.4%)	49 (75.38%)	1.3 (0.5, 3.8)
Gabapentinoid	100	63 (63%)	22 (62.9%)	41 (63.1%)	0.9 (0.3, 2.2)
NSAIDs	100	22 (22%)	5 (14.3%)	17 (26.2%)	1.9 (0.6, 6.4)
Acetaminophen	100	1 (1%)	0 (0%)	1 (1.5%)	Not estimatable
Psychotropic drug*	100	21 (21%)	8 (22.9%)	13 (20.0%)	0.8 (0.3, 2.4)

NSAIDs; non-steroidal anti inflammatory drugs

Age(group), sex, smoking, education (group), injury severity scale score (group), abbreviated injury scores chest (group) was adjusted for group comparison.
